# Tourists’ Thermal Experience and Health in a Commercial Pedestrianized Block: A Case Study in a Hot and Humid Region of Southern China

**DOI:** 10.3390/ijerph16245072

**Published:** 2019-12-12

**Authors:** Lei Zhang, Xuan Ma, Jingyuan Zhao, Mengying Wang

**Affiliations:** 1 Department of Architecture, Chang’an University, Xi An 710000, China; zl.wc@chd.edu.cn (L.Z.); zjyqtt@163.com (J.Z.); 2 Graduate school of Human-Environment Studies, Kyushu University, Fukuoka 8190379, Japan; 3HE18401S@s.Kyushu-u.ac.jp

**Keywords:** commercial pedestrianized block, outdoor thermal calendar, field measurement, numerical simulation

## Abstract

With the development of the economy in China, the tourism industry has become a form of daily entertainment for citizens. Commercial pedestrianized blocks have been designed as recreational centers for tourists, serving as outdoor public space and scenic spots. The use of these regions is directly determined by the outdoor thermal environment. So far, few studies have been conducted on tourists’ thermal experience in commercial pedestrianized blocks, especially in the hot and humid region of southern China. Using field measurement and numerical simulation of a commercial pedestrianized block in Fo Shan, China, to research tourists’ thermal experience under different conditions, the final results of this study could help to select the most suitable time for tourist travel and help local managers to improve the thermal environment.

## 1. Introduction

As the economy develops in China, tourism has become the most important source of entertainment in citizens’ daily lives. In addition, tourism is also a very important factor in the increasing employment and income of many cities in China [1]. Notably, the climate and weather in different seasons will directly affect the tour schedules of tourists; considering tourists’ thermal experience is very necessary, especially in extreme summer conditions. A suitable thermal environment could increase the number of tourists. When tourists are exposed to daytime sunshine that may cause thermal pressure, especially under extreme high temperatures, tourists’ health can be badly influenced [2]. The integration of the thermal environment and physical beauty has the potential to greatly improve tourist destinations [3]. 

When considering tourists’ thermal experience in different climates, choosing a suitable thermal index to analyze tourists’ thermal experience is necessary. To date, the discomfort index [4], wind-chill index [5], apparent temperature [6], and tourism climate index (TCI) [7] have been developed to evaluate thermal environments. These indices all consider various meteorological parameters, but consideration of the heat balance of the human body and human thermal physiology are still necessary. Therefore, based on former research, a new climate tourism information scheme (CTIS) index was developed to combine the energy balance of the human body and meteorological data [8]. Meanwhile, in accordance with the CTIS, some new indices to assess humans’ thermal comfort have been put forward, including standard effective temperature (SET) [9], effective temperature (ET) [10], universal thermal climate index (UTCI) [11], outdoor standard effective temperature (OUT-SET) [12], physiological equivalent temperature (PET) [13], and so on. The PET is based on the thermophysiological energy balance in the human body, which is also assessed by Germany’s VDI (Association of German Engineers) standard for its accuracy for calculating human thermal experience [14]. 

So far, some researchers have found that street morphology, including sky view factor (SVF) and aspect ratio (H/W), can affect the outdoor thermal environment [15,16,17]. The former is an index, ranging from zero to one, that describes the incoming daytime solar radiation, and the latter describes a proportional relationship between the width of a street and the height of the buildings. A higher H/W and lower SVF can contribute to improved outdoor thermal comfort. In addition, vegetation on both streets and rooftops can also ameliorate the thermal environment through evapotranspiration effects [18,19,20,21,22,23,24,25,26,27,28,29,30,31]. Reducing the percentage of hardened ground also can change inner microclimates [18,32,33]. 

Most previous studies on this aspect of tourism have been conducted in America [34] and Britain [35,36,37], Germany [38], the Netherlands [39], few studies has been conducted in southern China [1]. In addition, most studies to date have focused on the urban level, and very few have focused on the commercial pedestrian block. Due to its economic value, urban tourism has become a significant factor in the financial income of a city. Most previous studies on this topic have collected data from different meteorological stations and put forward thermal comfort conditions regardless of numerical simulation. In this study, besides the on-site measurements, a simulated ENVI-met tool was also used to evaluate outdoor thermal experience, thus creating a thermal calendar to help tourists schedule their trips and help managers and designers to understand the cooling effects of different strategies.

## 2. Methods

### 2.1. Research Site

**Figure 1:**
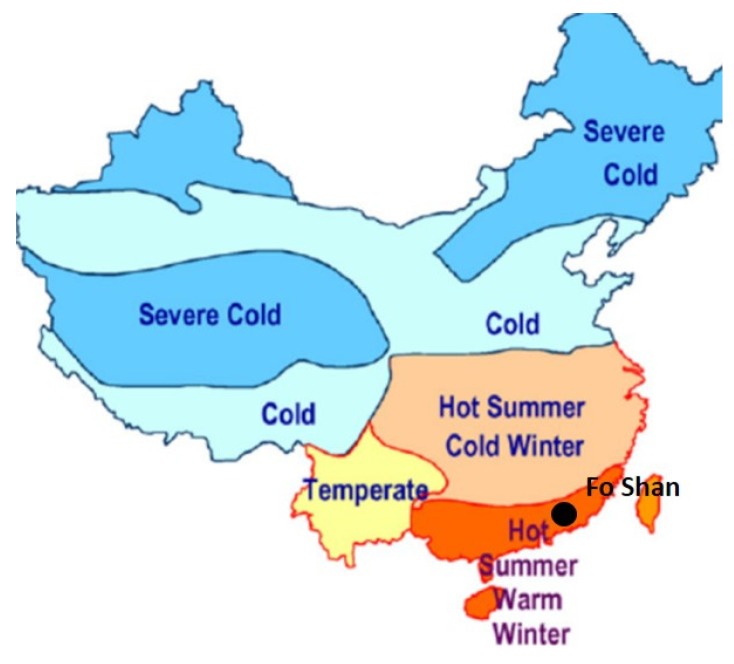
The climate classification of Fo Shan.

**Figure 2:**
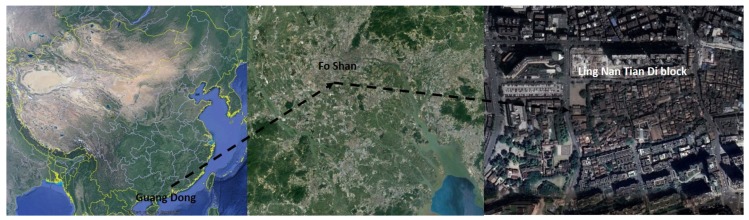
The location of the research site.

There are five classified climate zones in China: temperate zone, cold zone, severe cold zone, hot summer and warm winter zone, and hot summer and cold winter zone [40]. Fo Shan is a famous historical and cultural city in China, and also has a hot and humid climate in summer (hot summer and warm winter zone) (Figure 1). The Ling Nan Tian Di block is one of the most famous scenic spots in this city. According to the statistical data collected by local administration, it attracts many tourists every year (Figure 2) [41]. It is necessary to design a suitable environment for tourists.

### 2.2. Research Period

As mentioned above, the current study aimed to provide information about the most suitable traveling time for tourists. To this end, the researchers wished to assess the thermal environment both when there were the most tourists and the hottest period of a year. Based on the local published weather data, Fo Shan city experiences the highest air temperatures in July [42]. Table 1 shows the air temperature in July 2016.

In this study, on-site measurement was conducted at the research site to make sure the validation of the numerical simulation by ENVI-met was done at the same time as the on-site measurement. The final results were used to determine the outdoor thermal level of the selected site.

### 2.3. On-Site Measurement

**Figure 3:**
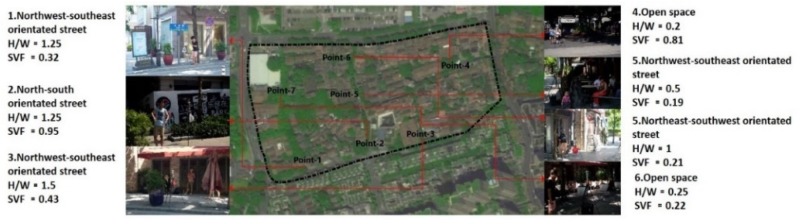
The selected points in this study.

Outdoor microclimate has a strong effect on tourists’ thermal comfort and affect their activity. This investigation conducts 7 points in accordance with the different geometry (Figure 3). In this study, the meteorological data including wind speed, air temperature and relative humidity are collected by the fixed instruments (Table 2).

Each point is measured for the typical day (hottest day, 24 July 2016), in addition, all the points are measured simultaneously from 9:00 a.m. to 5:00 p.m. The principles for fixing the instruments are as following:

1. Each instrument is fixed at a 1.5 m height (average pedestrian level) from the surface.
2. Each instrument is covered by a shelter to prevent the influence on air temperature by solar radiation at daytime.

### 2.4. Numerical Simulation

**Figure 4:**
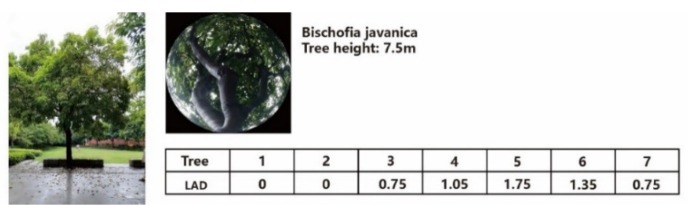
Vertical configuration of *Bischofia javanica*. LAD: leaf area density.

**Figure 5:**
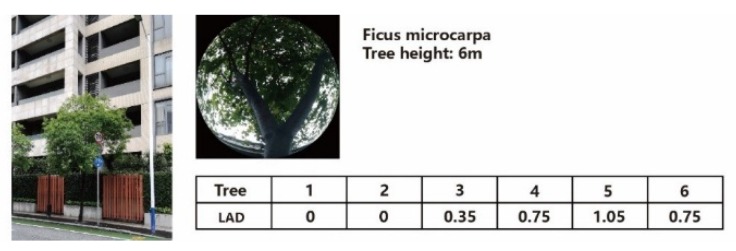
Vertical configuration of *Ficus microcarpa.*

As mentioned in many studies, numerical simulations can overcome the shortcomings of on-site measurement. In this study, the simulation was conducted using ENVI-met, which is a reliable tool for simulating outdoor thermal environments. As a point of difference from other software, the trees and grass in this software are set as biological bodies which can interact with the ambient environment by evapotranspiration. The configurations of the vegetation in this study were based on the leaf area density (LAD) and leaf area index (LAI), which is defined as a dimensionless value of the leaf area per unit of ground area, and explains the ability to impede incoming solar radiation. The following equation shows the relationship between the two [43,44]:

(1) $$LAI=\overset{\;h}{\underset{0}{\int }}LAD.\Delta z$$

where Δz is the vertical grid size (m) and h is the height of the tree (m). According to the field survey, the study region included two kinds of border tree, *Bischofia javanica*, and *Ficus microcarpa*. Detailed information on these two kinds of tree is shown in Figure 4 and Figure 5.

The initial input data used in this work are displayed in Table 3.

**Figure 6:**
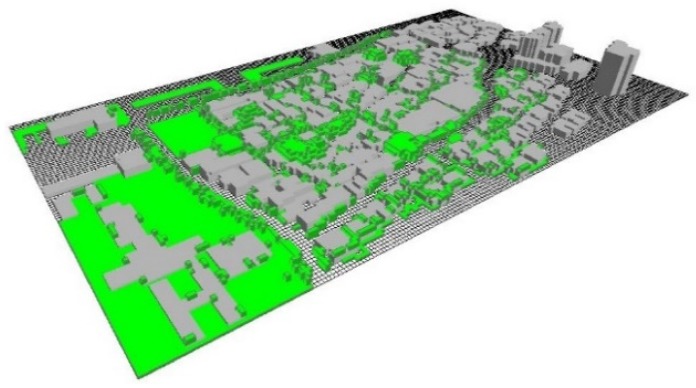
The simulated model of this study.

The simulated model of the selected site is shown in Figure 6.

The classification of thermal perception and PET values of the hot summer and warm winter climate zone is shown in Table 4 [45,46].

### 2.5. Validation

Even though the simulated accuracy of the ENVI-met has been tested in many studies, validation between the measured and simulated data was still necessary. In order to maximize the validity of the simulated results of this study, all the selected points of the simulation model were validated simultaneously.

**Figure 7:**
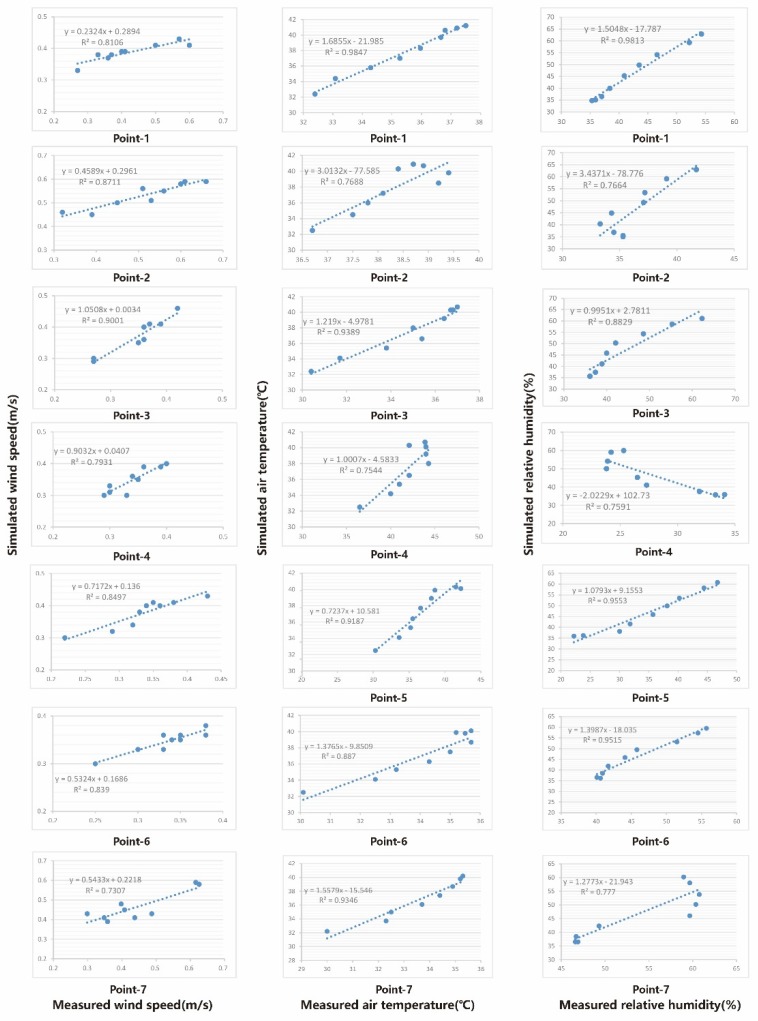
The correlation between three kinds of data [46].

The regression correlation [46] (Figure 7) of the R^2^ of the wind velocity was between 0.7307 and 0.9001, the R^2^ of the air temperature was from 0.7544 to 0.9847, and the values for relative humidity were between 0.7664 and 0.9813. The final linear regression results proved that the ENVI-met was a reliable software choice for this study.

## 3. Results

### 3.1. Tourists’ Thermal Experience under the Existing Scenario

**Figure 8:**
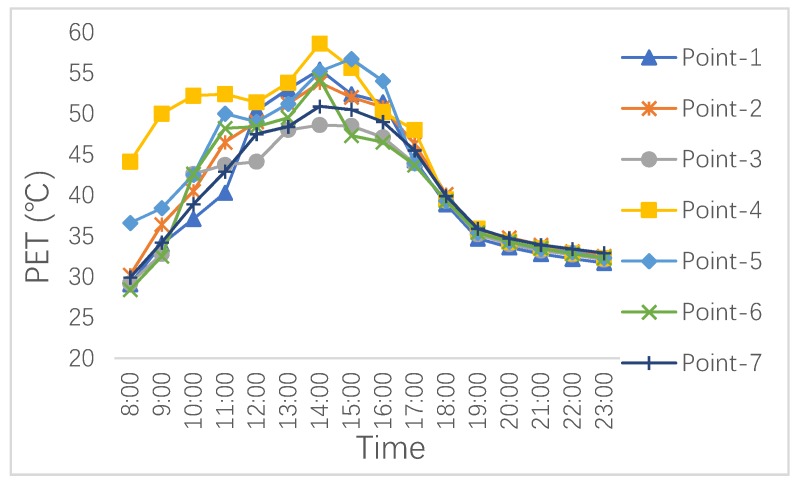
The thermal environment of the block under existing scenario [45].

As mentioned above, the PET index was used in this study to evaluate tourists’ thermal experiences in extreme summer. The thermal environment during the measured day is shown in Figure 8, which explains that the whole block reached “hot” and “very hot” levels from 10:00 to 19:00, meaning that nearly the entire day was unsuitable for tourist activities [45].

**Figure 9:**
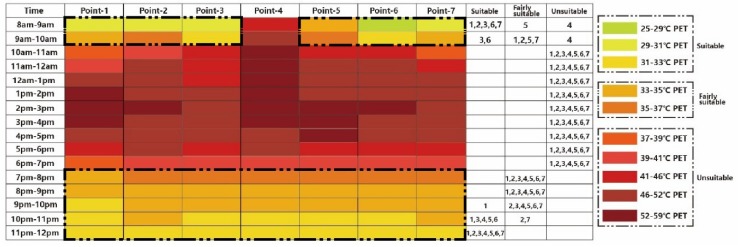
The thermal calendar under existing scenario [45].

Based on the existing thermal situation, a new thermal calendar has been put forward for tourists [45], which is in accordance with PET values and thermal perceptions of the hot summer and warm winter climate zone. In this calendar, each color represents a 2 °C interval of the PET value. “Very hot” and “hot” are defined as “unsuitable”, “warm” as “fairly suitable”, and “slightly warm” as “suitable”. Figure 9 shows the thermal comfort calendar under the existing scenario. As shown in this figure, from 8:00 to 9:00, nearly all the regions are at suitable and fairly suitable levels, except Point 4. From 10:00 to 19:00 p.m., the whole region is uncomfortable for visiting, and Point 4 has the worst thermal environment. After 19:00, all points can be easily visited.

### 3.2. New Thermal Calendars under New Cases

**Figure 10:**
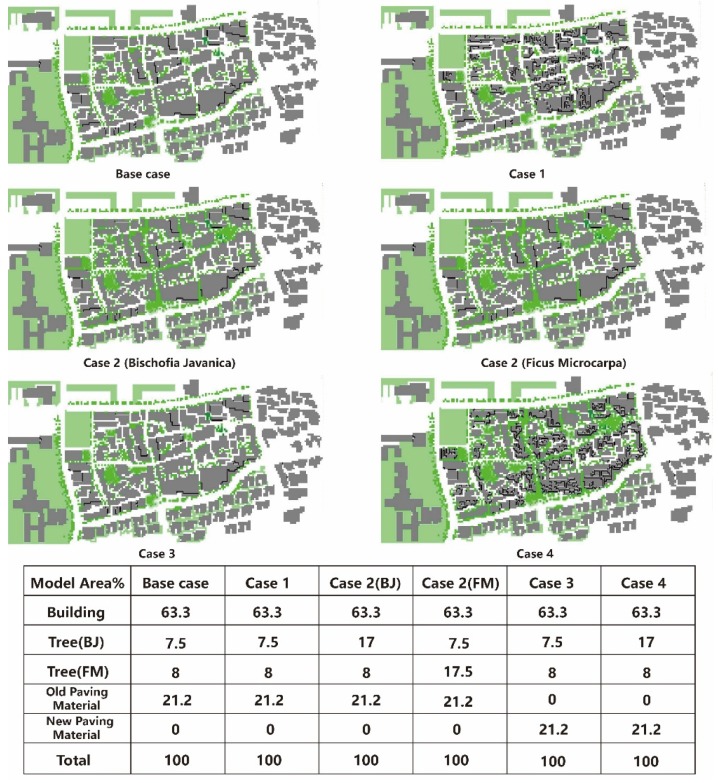
The cases: various different coverage ratios.

Based on the existing scenario, we put forward four new cases able to create a cooling effect and extend visiting hours. Table 5 and Figure 10 show the detailed information.

Case 1 aims at increasing the average building height, with its corresponding effect of improving thermal comfort. According to the local design specifications, the green coverage ratio in the built environment cannot be less than 25% of this whole region [47]. In Case 2, the tree coverage ratio is increased to 25%. Case 3 is focused on the cooling effect of a ground paving material with higher albedo. The last case combines all the mentioned cases together, and their functions were evaluated. As shown in Figure 10, in the base case (existing scenario), the total buildings occupy 63.3% of the whole region, the vegetation coverage ratios of *Bischofia javanica* and *Ficus microcarpa* are 7.5% and 8%, and the rest of the region (21.2%) is the ground surface. In Case 1, increasing the average building height cannot change the coverage ratio, so the results were similar to the base case. In Case 2, the coverage ratio of *Bischofia javanica* was increased to 17%, and that of *Ficus microcarpa* was increased to 17.5%. In addition, in Case 3, the ground surface was replaced with a higher albedo. In the last case, increasing the average building height and vegetation coverage ratio and replacing paving material were all applied.

**Figure 11:**
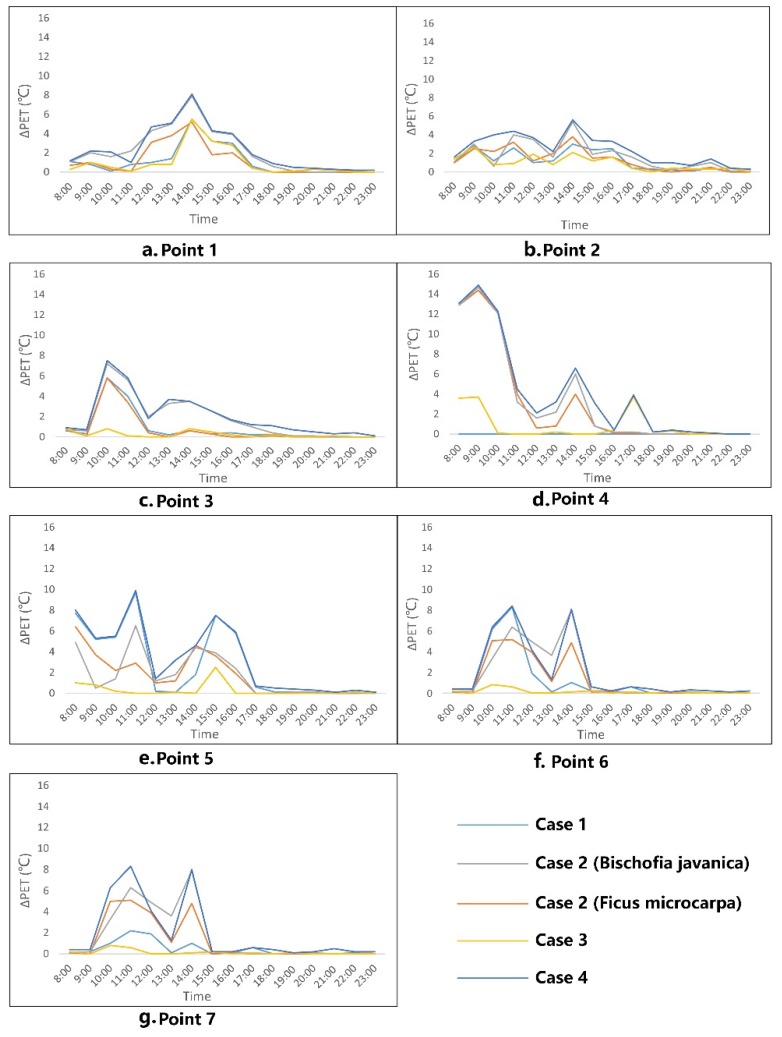
Thermal comfort improvement under different cases: (**a**) point 1; (**b**) point 2; (**c**) point 3; (**d**) point 4; (**e**) point 5; (**f**) point 6; (**g**) point 7.

Here, we discuss the impacts of the new cases on tourists’ thermal experience. The hourly diagram of ∆PET values of the selected points is shown in Figure 11. As expected, the magnitude of PET reduced under the new cases, that is to say, all new cases could contribute to improving outdoor thermal comfort (positive ∆PET). Figure 11a shows that for the ∆PET of Point 1, the last case (Case 4) has the strongest effect of reducing PET at daytime, with reductions ranging from 0.2 °C to 8.1 °C due to the building shadow and vegetation cooling effect. At daytime, the cooling effect of *Bischofia javanica* is much better than *Ficus microcarpa* because of the higher leaf area index (LAI); trees with higher LAI can better lower thermal experience through transpiration. Case 3 was not as effective as other cases at improving comfort. The ∆PET curve of Point 2 (Figure 11b) was similar to that of Point 1, however, the magnitude was less than that of Point 1. Because of the shared street orientation and similar geometry, the curve of Point 3 (Figure 11c) was similar to those of the aforementioned two points. For other canyon points (Figure 11d,e), the cooling effect of building shadow and vegetation was much better than for all aforementioned points. Under Case 4, the hourly ∆PET of Point 5 and Point 6 ranged, respectively, from 0.3 °C to 8.9 °C and 0.3 °C to 8.3 °C during the daytime. Case 3 was not effective at improving thermal comfort. For open space, the hourly variation of ∆PET is shown in Figure 11d,g. The time evolution of ∆PET at the two selected points was a little different; Point 4 had three peaks during the daytime, while Point 7 had two peaks. Moreover, unlike canyon space, Case 3 did not obviously reduce PET during the daytime. The effect of Case 4 on the two points was a respective reduction of PET by 14.9 °C and 8.6 °C.

**Figure 12:**
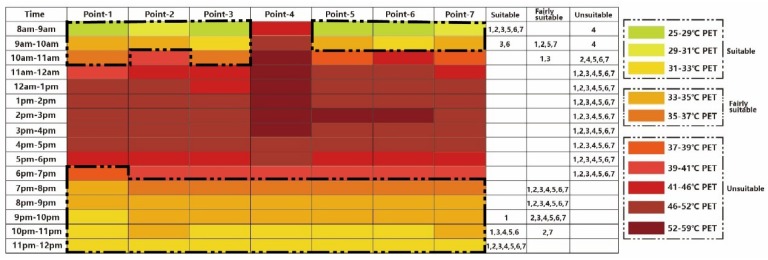
Thermal comfort calendar for tourists under Case 1.

In accordance with the new simulated outcome under increasing building height (Case 1), it was noted that the canyon with higher aspect ratio (H/W) (Figure 12) effectively reduced PET during the daytime. Unlike in the old thermal comfort calendar (base case) (Figure 9), from 8:00 to 9:00 in the morning, all points except Point 4 were suitable. Like in the existing scenario, after 19:00 all the points registered a good visiting environment.

**Figure 13:**
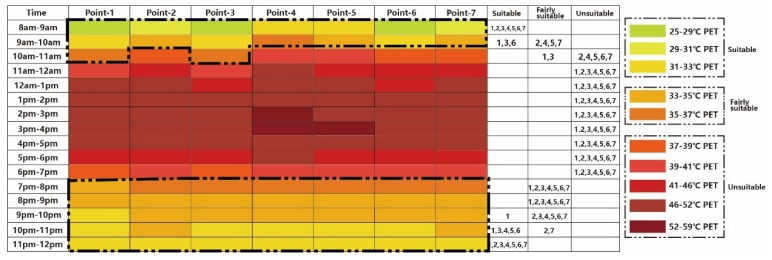
Thermal comfort calendar for tourists under Case 2 (*Ficus microcarpa*).

The simulated results showed that different tree species will have different cooling effects during the daytime. As Figure 13 shows, increasing the vegetation coverage ratio of this zone could broaden the visiting times for tourists compared to the existing scenario: Point 4 becomes able to be visited from 8:00 to 10:00, and Points 1–3 are all fairly suitable from 10:00 to 11:00. After 19:00, the difference between new thermal calendar and existing scenario is not obvious.

**Figure 14:**
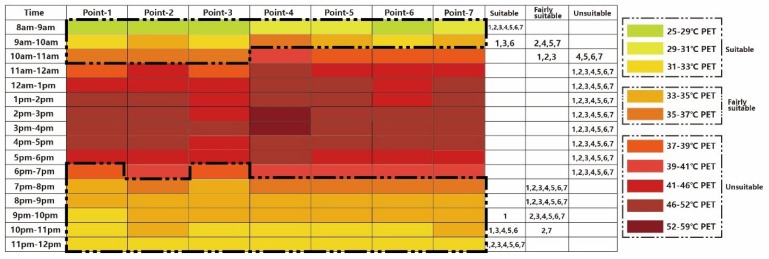
Thermal comfort calendar for tourists under Case 2 (*Bischofia javanica*).

As mentioned above, trees with a higher LAI will have a much greater effect on reducing PET during the daytime. As expected, the cooling effect of *Bischofia javanica* was much better than that of *Ficus microcarpa*. As shown in Figure 14, all the selected points were suitable from 8:00 to 9:00 in this case. Compared to the *Ficus microcarpa* scenario, even in the unsuitable period, *Bischofia javanica* can alleviate heat stress effectively.

**Figure 15:**
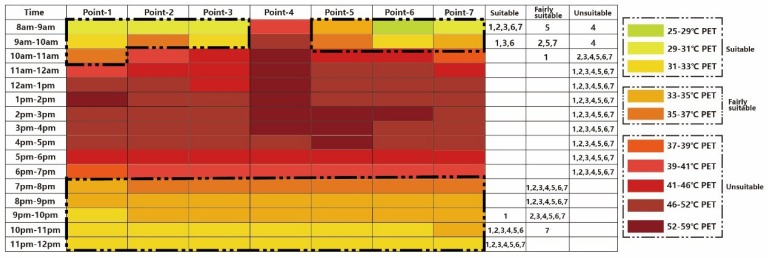
Thermal comfort calendar for tourists under Case 3.

Compared to the existing scenario, changing the paving material (Figure 15) did not effectively expand the cool time for tourists.

**Figure 16:**
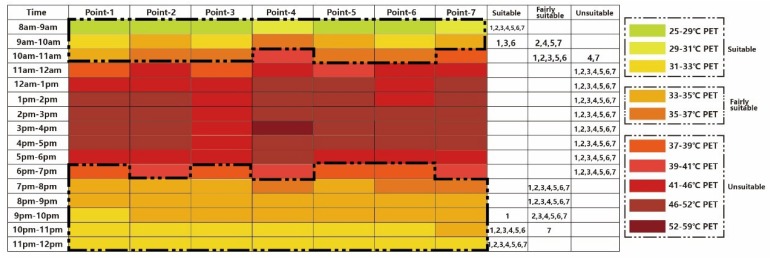
Thermal comfort calendar for tourists under Case 4.

Figure 16 shows the thermal calendar under Case 4. Compared to all the aforementioned calendars, the last case had the strongest influence on expanding the visiting time for tourists. Except for Point 4 and Point 7, other points could also be visited from 10:00 to 11:00.

## 4. Conclusions

The present study intended to evaluate tourists’ thermal experience in the microclimate of the valuable commercial pedestrianized-zone of Fo Shan city on the hottest day of a year. According to the simulated results under the existing scenario, almost none of the selected points were within the comfort zone during the daytime between 10:00 and 19:00. In addition, in the early morning (8:00 to 10:00), except for the open space (Point 4), other points were all in the comfortable zone. From 19:00 to 00:00, the whole zone is comfortable. The thermal calendars under the proposed new cases showed that increasing building height (Case 1) could effectively improve thermal comfort and expand the cool time for tourists in the canyon space, in which added nearly an extra hour for visiting. In Case 2 (*Ficus microcarpa*), the cooling effect not only improved human thermal comfort in the canyon space, but also in open space; in this case, the visiting time of the whole commercial region can be extended by an hour in the morning. Compared to Case 2 (*Ficus microcarpa*), Case 2 (*Bischofia javanica*) had a higher LAI, and the final result showed that it had a better cooling effect. Case 3 showed that the cooling effect of pavement material (a higher albedo of ground surface), even when it alleviated heat stress did not extend the visiting window by much. The final case (Case 4) showed the highest cooling effect, in which the visiting time for tourists could be extended by two hours in the morning. The final results could help tourists to choose a comfortable period for their visiting, and also provide urban planning recommendations.

Based on the conclusions of this study, our suggestions are: (1) Increased coverage ratio of three-story buildings. Increased average building height can effectively impede solar radiation during the daytime, which will always provide a benefit for outdoor environments. (2) The vegetation and landscape represent an influential factor; our final results proved that there is an obvious correlation between the trees and the reduction of PET values. In Fo Shan city, it is recommended to plant trees with a higher leaf area index (LAI) (*Ficus microcarpa*, *Bischofia javanica*, *Camphora officinarum*, Chinese redbud, *Carthamus tinctorious*, and so on). (3) Reduced percentage of hardened ground with lower albedo; designers can use paving materials with higher albedo to improve the outdoor thermal environment.

In addition, there were some limitations to our study. The simulated air temperature showed a 5.5 °C deviation from on-site data, which was attributed to the inability of the models to simulate aspects of reality like the façades of buildings, and the limited resolutions in ENVI-met. This study evaluated outdoor thermal comfort under the trees currently existing at the research site; future work should consider more vegetation.

## Figures and Tables

**Table 1:** The weather conditions on the measured days.

July	1	2	3	4	5	6	7	8	9	10
Weather	Rain	Rain	Rain	Rain	Rain	Rain	Rain	Rain	Rain	Rain
Min air temperature (°C)	26	27	27	26	26	26	26	28	29	28
Max air temperature (°C)	31	31	31	31	31	32	34	36	36	34
July	11	12	13	14	15	16	17	18	19	20
Weather	Rain	Rain	Rain	Rain	Rain	Rain	Rain	Rain	Rain	Rain
Min air temperature (°C)	27	26	27	27	27	28	28	28	27	27
Max air temperature (°C)	32	30	34	33	34	35	34	34	33	33
July	21	22	23	24	25	26	27	28	29	30
Weather	Rain	Cloudy	Sunny	Sunny	Sunny	Sunny	Rain	Cloudy	Cloudy	Rain
Min air temperature (°C)	26	26	26	27	27	27	27	26	27	28
Max air temperature (°C)	34	36	36	37	37	36	34	35	37	38
July	31									
Weather	Rain									
Min air temperature (°C)	27									
Max air temperature (°C)	35									

**Table 2:** The introduction of the measured instruments.

Instrument	Mode	Accuracy	Range	Interval	Sensor
Wind speed	Automatic	±0.3m/s	0–70m/s	60s	DS-2
Relative Humidity	Automatic	±5%RH	10–95%RH	60s	TR-70wf
Air Temperature	Automatic	±0.5 °C	0-+55 °C	60s	TR-70wf

**Table 3:** Initial data for simulation.

Initial Data	Content
Beginning time	0:00, 24 July 2016
Total time	24 h
Roughness length	0.1
Air temperature	38 °C
Relative humidity	45%
Wind velocity in 10 m	1.8 m/s
Wind direction	145°
Albedo of wall	0.3
Albedo of roof	0.2
Albedo of ground	0.4
Dimension of the grid in dx	3 m
Dimension of the grid in dy	3 m
Dimension of the grid in dz	2 m
No. of x grid	200
No. of y grid	100
No. of z grid	30

**Table 4:** The physiological equivalent temperature (PET) values and humans’ thermal perceptions [45,46].

PET (°C)	Thermal Perception
<13	Very cold
13–17	Cold
17–21	Cool
21–25	Slightly cool
25–29	Neutral
29–33	Slightly warm
33–37	Warm
37–41	Hot
>41	Very hot

**Table 5:** The new cases.

Case	Introductions
1	Increasing average building height
2	Increasing tree coverage
3	Replacing the paving material with higher albedo (ground albedo = 0.6)
4	Case 1 + Case 2 (Bischofia Javanica) + Case 3
